# Search Engine Gender Bias

**DOI:** 10.3389/fdata.2021.622106

**Published:** 2021-05-26

**Authors:** Fons Wijnhoven, Jeanna van Haren

**Affiliations:** Department of Industrial Engineering and Business Information Systems, University of Twente, Enschede, Netherlands

**Keywords:** personalization, Google, DuckDuckGo, filter bubble, gender bias, job search, political participation search

## Abstract

This article discusses possible search engine page rank biases as a consequence of search engine profile information. After describing search engine biases, their causes, and their ethical implications, we present data about the Google search engine (GSE) and DuckDuckGo (DDG) for which only the first uses profile data for the production of page ranks. We analyze 408 search engine screen prints of 102 volunteers (53 male and 49 female) on queries for job search and political participation. For job searches *via* GSE, we find a bias toward stereotypically “female” jobs for women but also for men, although the bias is significantly stronger for women. For political participation, the bias of GSE is toward more powerful positions. Contrary to our hypothesis, this bias is even stronger for women than for men. Our analysis of DDG does not give statistically significant page rank differences for male and female users. We, therefore, conclude that GSE’s personal profiling is not reinforcing a gender stereotype. Although no gender differences in page ranks was found for DDG, DDG usage in general gave a bias toward “male-dominant” vacancies for both men and women. We, therefore, believe that search engine page ranks are not biased by profile ranking algorithms, but that page rank biases may be caused by many other factors in the search engine’s value chain. We propose ten search engine bias factors with virtue ethical implications for further research.

## Introduction

Search engines enable their users to receive relevant search results in the enormous mass of data on the Web. The most used and best known search engine is the Google search engine, named GSE in the rest of this article. By collecting data about its users, GSE creates personal profiles of its users for targeted advertising service (Thurman and Schifferes, 2012). With the same process, GSE also personalizes search outcomes and page ranks for optimizing a search outcomes relevance experience for its users (Rogers 2013; De Corniere and Taylor, 2014). Personal profile processing, as part of search query processing, may result in a high use value experience but also can keep important less relevant information away from its user (Helberger et al., 2018). This “filter bubble effect” (Pariser 2011) also comes with benefits, as people lack the capacity to process all the zettabytes of information available on the Internet. This also implies that people with different profiles who enter the same query into the GSE receive a different list of relevant Web pages. This ability of semantic search enables GSE to differentiate the meaning of ambiguous terms in a query. For example, while a lawyer would refer to the keyword “code” as a set of rules or the law, an information technology specialist may be more interested in “code” referring to software, and thus a profile aware search engine will serve both differently. This subjective relevance increasing process may, in a subtle way, result in influencing users to certain viewpoints and opinions (Epstein and Robertson 2015), which is a process also named nudging (Gal et al., 2020).

A common nudge in computer environments is the setting of defaults, which are preset courses of action that take effect if nothing is specified by the decision maker (Cronqvist et al., 2018). But nudging can also take more elaborate and sinister forms. For example, in 2017, it became known that advertisers on Facebook could target advertisements at teenagers during moments of psychological vulnerability. This kind of nudging can also be employed by organizations through the use of algorithmic systems to encourage “appropriate” behaviors. For instance, Kaptein et al. (2015) have shown that emails and Web site usage nudges can move people to more healthy behavior. But these practices can also be manipulative and ethically questionable when they are aimed at covertly subverting people’s decision-making capacity by exploiting their psychological, cognitive, or emotional vulnerabilities to change their beliefs, thoughts, or behaviors. Such manipulative practices limit people’s capacity for voluntary action and learning. Nudging may not only be unethical from a utilitarian perspective (Tavani 2012), that is, the diminishing value of what is received for the search engine user, but also from a virtue ethics perspective as it disallows the user to identify the source of bias, learn from that, and reduces the freedom of voluntary action of what to believe or not (Gal et al., 2020). Following Gal et al. (2020), we choose for a virtue ethics approach for the analysis of search engine bias. Virtue ethics, based on the philosophical work of Aristotle, highlights personal characteristics in determining the ethical nature of individuals and their actions. Virtue ethics focus on the virtuous agent rather than on the right actions to reach certain values (i.e., the utilitarian approach) or on what anyone should do given certain rules (i.e., the deontological approach to ethics).

The ranking of search results is especially important because users tend to pay more attention to the results that rank higher on the search results page (Bozdag 2013). The actual impact of search engine and page rank biases is also named as the search engine manipulation effect. A slight manipulation of a page rank can have an impact on the outcome of elections by providing search results that are in favor of one candidate (Epstein and Robertson, 2015). This eventually causes a user to be less likely to discover new topics on the Web or to see documents with different views on a topic that contradicts his or her values (Wilson et al., 2012; Burger et al., 2016). The purpose of this study was to gain further insights into possible search engine bias toward one-sided search results with ethical implications.

This leads to the research question: To what extent does the Google search engine creates a biased page rank for its users with negative virtual ethical consequences?

To answer this question, this article focuses on search engine gender bias and its virtue ethical implications for women. Besides the commitment of the authors to equal opportunities of women in our society, gender characteristics are one of the most common and easily traced search engine profile features (Kosinski et al., 2013). A search engine’s gender bias is the influence of gender features in a search engine profile on gender bias of a search engine’s outcomes and page rank (Lopes et al., 2016). The insight from a gender-bias study can produce generalizable understandings on how search engines may produce, reproduce, or reinforce biases with virtue ethical implications for other social segments.

## Background, Theory, and Hypotheses

Personalized search results are customized search results based on the digital profile of an individual user. When using a personal profile aware search engine such as GSE or Bing, search results will differ depending on who is supplying the query to the search engine. This profile is supposed to represent the interests of the individual user (Harvey et al., 2013). GSE’s updated privacy policy of 2012 states that information is being shared on all sorts of Google tailors, which include Gmail, YouTube, and Google Maps for further profiling of the search engine user (Voss 2014). Also, search location indicators (Hannak et al., 2013) and browsing histories can combine with user attributes to produce personalized outcomes (Goel et al., 2012). A manipulation effect occurs if the search engine can influence users’ attitudes, beliefs, or decisions based on the order of the search engine results. A manipulation effect may result from search results that are biased toward one view. By providing one-sided search results, GSE could either reinforce an existing opinion or it may turn an undecided person to be nudged toward one opinion direction (Epstein and Robertson 2015; Wijnhoven and Brinkhuis 2015).

There is a substantial volume of research outcomes regarding the difference in the use of the Internet between men and women (Colley and Maltby 2008; Yom-Tov and Elad, 2019), but there is little knowledge on what the role of the medium is in this story. Hargittai and Shafer (2006) suggest that the supply-side of content—due to its structure and presentation—is in itself male-biased. This would imply that gender bias of search engine page ranks is not necessarily the result of personalization but could be the result of biased supplies of information on the Internet as well. Unfortunately, it is difficult to identify what GSE knows about you, we only know that personal profile processing is a key part of GSE’s proprietary way of delivering relevant content to its user (Bozdag 2013; Rogers 2013). Such a personal profiling algorithm is lacking in privacy-aware search engine DuckDuckGo (DDG), and thus comparing page ranks of male and female users of both using GSE and DDG for the same queries would be able to determine if the search engine is the cause of page rank bias or if alternative reasons exist for page rank bias. For knowing if this bias has virtue ethical consequence, we will take queries that may have important consequences for women to learn and develop themselves.

This section first goes into more details about causes and consequences of search engine bias. Then it will set out the concerns regarding gender inequalities and our hypotheses.

### Causes and Virtue Ethical Consequences of Search Engine Bias

Search engine bias includes a selective presentation of Web documents to a search engine user. This selectivity can be selectivity of sources, content, views, and page ranks (Bozdag 2013). The causes for these biases can be explicit by censorship that disables users to access certain sources, content, viewpoints, and algorithms, that is, will make it difficult for users to find and see certain content, or the outcome of optimization actions of content suppliers, that is, through search optimization. Finally, search engine bias may also be the result of account and behavior information that is delivered to the search engine, and by which the search aims at increasing its service level may increase in the perception of the user. If this user profile information is used for page ranking, it increases the probability that users will access less diverse sources, content, and viewpoints, which is called the filter bubble (Pariser 2011; Flaxman et al., 2016).

Page rank bias has ethical implications, because search engine outcomes may impact people’s opinions, choices, and actions, and search engines may be intentionally used by stakeholders to influence society by attempts of censorship and search engine optimization (Fu and Karan 2015; Baye et al., 2016). The influence of search engine should not be understated and thus identifying possible page rank biases is important from an ethical perspective (Flaxman et al., 2016; Haim et al., 2018), and its page rank outcomes can nudge people toward certain opinions and decision (Epstein and Robertson 2015) and reinforce possible stereotypical views on people and gender (Kay et al., 2015; Otterbacher et al., 2017). Recent experiences have shown that GSE sometimes intentionally nudges its users toward Web pages that are commercially more interesting from Google’s own perspective (Blanckenburg 2018). Google has been punished for this by the European Committee and corrections have been implemented, but also for nonshopping purposes, search biases can easily happen because of a lack of transparency and the proprietary nature of GSE services (van Drunen et al., 2019). The virtue ethical implications of search engines can be very diverse. For example, it may result in a lack of capability of voters to judge and choose among election candidates (Epstein et al., 2017), or the reinforcement of racist views and behavior (Noble 2018), and the lack of critical thinking (Heersmink 2018). We choose for a focus on virtue ethics from a gender perspective, that is, the abilities of women to emancipate from stereotypical roles and achieve gender equality on the workplace and society.

### Gender Inequalities

According to the United Nations Development Program (UNDP, United Nations Europe and Central Asia, 2018), there are still significant inequalities between women and men in Europe and Central Asia, particularly when it comes to jobs and income, political participation, and the distribution of unpaid domestic and care work. Similarly, gender stereotypes are prevalent, hindering women’s access to opportunities. Furthermore, men are more likely to gain promotion to top management positions and prestigious leadership roles than women. Moreover, women are more likely to have insecure jobs, no contract or regular salary, or part-time jobs (UNDP, United Nations Europe and Central Asia, 2018). Search engine outcomes, like any message or piece of information, can reproduce or reinforce views on and the reality of gender inequalities related to jobs and leadership roles in society (Otterbacher et al., 2017; Chen et al., 2018). We, therefore, will go in more detail on jobs and leadership roles in society as two gender-specific virtue ethical topics.

#### Jobs

According to Scherer (2004), the labor market entry and a successful transition from school to work are of crucial importance for subsequent career chances, it is a good start to look at the possibly different opportunities here for men and women. Defloor et al. (2015) found that the quality of a person’s first job is largely dependent on personal effort, although circumstances, for example, gender do have a considerable influence on these efforts. This would, therefore, suggest that a difference of jobs between men and women is due to a difference in effort spent between men and women when searching for their first job. Equality of opportunity besides efforts is also highly debated in relation to gender. Equality of opportunity means that outcomes experienced by a population depend only on factors for which persons can be considered to be responsible (Roemer and Trannoy 2015). As people are increasingly searching for jobs online (Nikolaou 2014), gender indicators in personal profiles may disturb the equality of finding the same job opportunities on the Internet, although Chen et al. (2018) did not find evidence for gender bias in recruiting site algorithms. As Pariser (2011) pointed out, the filter bubble not only reflects a person’s identity but it also selectively shows the related options a person has. This may be positive, because some options may be less relevant for people with a specific profile, for example, jobs for university alumni have less relevance for those with higher vocational education. In defense of personal profile processing, one may see this as the effective working of relevance. Alternatively, however, personal profile processing search engines can replicate and reinforce current gender stereotypes on the job market. The existence of these stereotypical outcomes as a consequence of the usage of user profile information has been identified before by Otterbacher et al. (2017), but one may ask the question if a privacy-aware search engine like DDG will be free of such bias. Table 1 gives a list of various occupations classified as male-dominated, mixed, or female-dominated based on data from 12 EU countries.

**TABLE 1:** Gender occupation stereotypes (Bettio and Verashchagina 2009).

Occupation	Number of countries where occupation is dominantly…		
	Male	Mixed	Female
Armed forces	10	0	0
Legislators, senior officials, and managers	7	5	0
Corporate managers	8	4	0
Managers of small enterprises	5	7	0
Physical, mathematical, and engineering science professionals	12	0	0
Life science and health professionals	0	7	5
Teaching professionals	0	2	10
Other professionals	0	11	1
Physical and engineering science associate professionals	11	1	0
Life science and health associate professionals	0	0	12
Teaching associate professionals	0	2	8
Other associate professionals	0	10	2
Office clerks	0	0	12
Customer services clerks	0	0	12
Personal and protective services workers	0	2	10
Models, salespersons, and demonstrators	0	1	11
Skilled agricultural and fishery workers	8	4	0
Extraction and building trades workers	12	0	0
Metal, machinery, and related trades workers	12	0	0
Precision, handicraft, craft printing, and related trades workers	5	7	0
Stationary plant and related operators	11	1	0
Machine operators and assemblers	5	6	1
Drivers and mobile plant operators	12	0	0
Sales and services elementary occupations	0	0	12
Agricultural, fishery, and related laborers	3	6	2
Laborers in mining, construction, manufacturing, and transport	12	0	0

Furthermore, regarding working sectors, women make up almost 80% of those employed in health and social work, over 70% of those employed in education, and over 60% of those working in retailing. In contrast, only 8% of those employed in construction and 14% of those in land transport are women. This pattern is visible throughout all EU member states. We thus expect the following from a profile-aware search engine:

H1: Women are mainly shown vacancies for female-dominant jobs in their GSE search results.

H2: Men are mainly shown vacancies for male-dominant jobs in their GSE search results.

#### Political Participation

Cabeza-García et al. (2018) found differences between men and women regarding political participation. They found evidence that participation in bodies of power and institutions is predominantly masculine, whereas voluntary associations, organizations, and “informal” community politics tend to be led by women. Moreover, the number of women as member of governments was only 16% in 127 countries, and not one of the women was in a top parliamentary management post. Furthermore, the women who had a post in local, municipal, or national government occupied posts with a more social and cultural nature and thus with less political importance.

Brandtzaeg (2017) studied the difference between men and women in expressions of civic engagement on Facebook and found that millennial women more often engage with posts related to children and the environment when compared to men of the same age. Moreover, it was found that patterns in the offline world regarding the differences among men and women in civic engagement were reproduced and reinforced rather than equalized on Facebook.

Pfanzelt and Spies (2019) studied the difference between young German men and women regarding political participation. They distinguished between three types of political participation, namely, institutional, noninstitutional, and expressive. The first one covers activities that mainly address the state *via* participation in elections or actively running for or holding office. Noninstitutional participation refers to protest activities like boycotts and expressive participation includes giving voice to political aims and intentions of citizens. The Internet is a main medium for expressive participation. They found that young men are more likely to participate in institutional and expressive forms, while young women tend toward noninstitutional, protest-oriented activities.

As explained above, women are more likely to engage in certain political practices while men in other, which could possibly be reflected and reinforced through personalization techniques used by GSE. We believe that this difference between men and women in politics can be interpreted as involvement of women in less powerful political activities and involvement of men in more powerful activities, consistent with previously identified stereotypes of competent men and warm women as found on an analysis of Internet images (Otterbacher et al., 2017). This leads to the following hypotheses:

H3: Women are mainly shown political involvement opportunities for positions of low power and influence in their GSE results.

H4: Men are mainly shown political involvement opportunities for positions of high power and influence in their GSE results.

### DuckDuckGo

DuckDuckGo (DDG) is said to not gather personal data from its users for its page ranks (Hannak et al., 2013). Instead, DDG bases their search results on expert advice. This means that they focus only on the search term and its semantics and what documents an expert would recommend to access (Jansen and Spink 2006; Jatwani et al., 2020). Thus, considering the method used by DDG, it is expected that search results for every individual user will be nearly the same. This leads to the following hypotheses:

H5: There is no gender bias in the vacancies shown by DuckDuckGo to men and women.

H6: There is no gender bias in the political involvement opportunities shown on DuckDuckGo to men and women.

## Methodology

This section describes the methodology for observing and analyzing if people’s search results are biased.

### Research Design

DDG and GSE are the units of analysis in this study. In total, 144 students participated in this study. All participants needed to be at least 18 years of age to assure that they are legally allowed to accept the terms of this research study and that their data are being used for research purposes. Participants should use GSE as their main search engine and should have also not removed their browser cookies too recently so that a rich personal profile could be created by GSE. Participants have been excluded from the research if they have removed their browser cookies in the last 2 weeks. Furthermore, individuals need to participate in the experiment on their own personal laptops or computers to be suitable for the study.

Two search queries have been used that address issues that can cause people to have different page ranks. The selected search queries are about highly different gender virtue issue to avoid possible carry over effects, that is, one search query influencing another one in the same study.

### Data Collection

Regarding the gender issues, participants of the survey are asked to type in the two search queries into GSE and the same two into DDG. These two search queries are “job openings near me” and “how to become involved in politics.” These two search queries are based on the literature about the gender gap in occupations and political participation as described in *Background, Theory, and Hypotheses* section before.

To be able to measure the construct of bias, the study consisted of five parts. The first part asked questions to determine the suitability of a participant. The second part asked general questions regarding demographics and behavior on GSE of the respondent, for example, age, educational level, and the language mostly used to conduct searches. The third and the fourth parts asked the participants to upload screenshots of their first six organic search results appearing, when searching with “job openings near me” and “how to become involved in politics.” These organic search outcomes are the nonpaid search outcomes of the search engine, normally found below the paid search outcomes (i.e., advertisements) of the search engine. The third part of the study asked this for GSE, while the fourth part asked to do this for DDG. The fifth part is the analysis of the results.

To gather enough participants, the link of the answer form has been shared on the personal Facebook page of two of the author’s students who also participated in the coding of the outcomes, one of them who is also the co-author of this article. Furthermore, it has been shared in the WhatsApp groups of these two students.

### Participants’ Actions

We received 144 respondents; however, not all of these respondents could be used due to certain requirements set for this research. One of these requirements is related to cookies. Participants were asked when they removed their cookies for the last time. A cookie is a small piece of data sent from the Web site and stored on the user’s browser that is sent back to the Web site every time the user returns (Coey and Bailey 2016). The information stored on the cookies is used to create a user’s profile by GSE. This has led to the decision of including all participants who removed their cookies two weeks ago or later as the frequent use of the Internet and the instant installation of cookies gives GSE an impression of the users’ profiles in these 2 weeks. Other requirements include the use of a personal computer or laptop instead of a phone, being 18 or older, and the use of GSE as the main search engine. This left 102 suitable participants for this study, 53 males and 49 females. All of the participants are students following a higher vocational education, bachelor, or master. The age ranges from 18 to 29 years, with most participants in the range of 20–23 years. The three most occurring nationalities are Dutch (60.8%), Mexican (8.8%), and German (7.8%).

### Analysis

To analyze the collected data, a coding scheme is designed for determining if a query result is biased toward one side. The data analysis in this research is a directed content analysis because this research has started with an explanation of theories related to the research question and from this the coding scheme has followed (Hsieh and Shannon 2005). Only the first four delivered screen shots of organic search results have been analyzed, as the fifth and the sixth result were not always visible on all screenshots. This makes a total of 408 screen shots to be coded. The points given to a screenshot range from −6, “only male-biased search results are shown,” to 6, “only female-biased search results are shown.” The first two results have been given extra weight and score −2 for male-biased and 2 for female-biased, as according to (Epstein et al., 2017) people generally believe that the top results are presented first. The third and the fourth result are given either −1, for male-biased, or 1 for female-biased. This way, this study measures page ranking bias. If there is no bias present, zero points are given. For example, when the first three search results were determined to be female-biased and the fourth was determined male-biased, the screenshot would receive a score of 2 + 2 + 1 − 1 = 4. The detailed coding scheme is given in Supplementary Appendix Tables S1, S2.

After the data have been coded, it has been statistically analyzed. Independent-samples *t* tests have been chosen for comparing the statistical significance of mean for the two GSE and DDG male and female users (sub)samples. One-sample *t* tests are conducted for determining the differences of the means relative to neutral values for the GSE and DDG page rank outcomes. Both tests require the data to be normally distributed and therefore for all four variables (“Jobs Google,” “Politics Google,” “Jobs DDG,” and “Politics DDG”), a Shapiro–Wilk test has been conducted to test for normality. The Shapiro–Wilk test uses the null hypothesis that the population is normally distributed, which is rejected whenever the *p*-value falls below a certain alpha value, in our case when the *p*-value is lower than 0.05. For all four variables, the null hypothesis of normal distribution is rejected as the *p*-value is below 0.05. Hence, a further analysis of Q-Q plots is used to check the normality of the data distribution by a visual inspection of the line that the data make with the theoretically ideal normal distribution. These plots show straight upward lines, thus accepting the data for further analysis (Jaccard and Becker 2002) (data are available on request).

### Reliability

The two persons who helped in the data collection independently performed the coding of the screenshots after discussing the coding scheme of Supplementary Appendix Tables S1, S2 with the first author. For testing the interrater reliability of the coding, we used Krippendorff’s alpha (Hayes and Krippendorff 2007). Krippendorff’s alpha is a statistical measure that displays the extent of agreement between coders on the values given to a variable on the basis of a coding scheme. This test is suitable for ratio data which we collected for this study. With the scores assigned to the screenshots a meaning is given to the data ranging from −6 (fully male-biased) to 6 (fully female-biased), the intervals are of equal distance and there is an absolute zero point as assigning a zero means that there is no bias. Before checking for reliability, the results of coding for GSE and DDG have been combined per variable. This has been done because the same coding scheme has been used for “Jobs Google” and “Jobs DDG” as well as for “Politics Google” and “Politics DDG.” For both categories, Krippendorff’s alpha were higher than 0.67, namely 0.7682 for “Jobs” and 0.7725 for “Politics,” which means that the coding schemes for both political participation and jobs were sufficiently reliable (Hayes and Krippendorff 2007).

## Results

We present the results for the GSE and DDG jobs and political participation queries per hypothesis in the following subsections.

### Jobs GSE (H1 and H2)

A one-sample *t* test has been conducted to test if there is a bias toward either side. The results differ significantly from zero (one-sample *t* test = 6.286, df = 96, *p* < 0.000), meaning that the search results for “job openings near me” are biased toward one side as zero means neutral results. This bias is in the direction of female-dominated jobs as the average score of the screenshots is 1.36.

With the coding scheme used in this study, the hypothesis is that women will have a significantly higher score than men. An independent-samples *t* test supports this first hypothesis: “women are mainly shown vacancies for female-related jobs in their GSE search results” (independent-samples *t* test = −1.845, df = 81.795, *p* = 0.0345). Surprisingly, the mean score of men is 0.98, which also implies that on average men were shown more female-dominated jobs instead of male-dominated jobs. Before being able to say with evidence that the second hypothesis is not supported, a one-sample *t* test only for men has been conducted. The results from men are indeed significantly different than zero (one-sample *t* test = 3.951, df = 50, *p* < 0.000). This means that they are shown one-sided views on the search query “job openings near me”; however, it was not in the expected direction. Therefore, the second hypothesis is not supported. The SPSS output for these tests is given in Table 2.

**TABLE 2:** SPSS output for “Job openings near me Google.”

Sample statistics for “Job openings near me Google”				
		Mean	Std. deviation	Std. error mean
N	97	1.36	2.132	0.216
Male	51	0.98	1.772	0.248
Female	46	1.78	2.421	0.357

**One-sample test**					
**T**	**Df**	**Sig. (2-tailed)**	**Mean difference**	**95% confidence interval of the difference**	
								**Lower**	**Upper**
6.286	96	0.000	1.361	0.93	1.79

**Independent-samples test**									
	**Levene’s test for equality of variances**					***t*** **test for equality of means**		**95% confidence interval of the difference**	
**Equal variance**	**F**	**Sig.**	**t**	**Df**	**Sig. (2-tailed)**	**Mean difference**	**Std. error difference**	**Lower**	**Upper**
Assumed	7.757	0.006	−1.875	95	0.064	−0.802	0.428	−1.652	0.047
Not assumed			−1.845	81.795	0.069	−0.802	0.435	−1.667	0.063

**One sample test including only male participants**					
**T**	**Df**	**Sig. (2-tailed)**	**Mean difference**	**95% confidence interval of the difference**	
								**Lower**	**Upper**
3.951	50	0.000	0.980	0.48	1.48

### Political Participation GSE (H3 and H4)

Starting with a one-sample *t* test to determine if there is a bias to be found in the search results for the query “how to become involved in politics,” the test gives that the results differ significantly from zero (one-sample *t* test = −6.872, df = 92, *p* < 0.000). The general bias is in the direction of political positions with high power and influence, as the mean is −1.89. Hypothesis 3 states that women would have significantly higher scores than men and that women would have a positive score. This hypothesis is rejected because women have a mean score −2.30, even higher than men who have an average score of −1.48. Clearly, men have a negative score, as expected, and hypothesis 4 is not rejected, but the *t* test has no significant difference between men and women (independent-samples *t* test = 1.498, df = 91, *p* = 0.069). The SPSS output is given in Table 3.

**TABLE 3:** SPSS output for “How to become involved in politics Google.”

Sample statistics for “How to become involved in politics Google”				
		Mean	Std. deviation	Std. error mean
N	93	−1.89	2.656	0.275
Male	46	−1.48	2.689	0.397
Female	47	−2.30	2.587	0.377

**One-sample test**						
**T**	**df**	**Sig. (2-tailed)**	**Mean difference**	**95% confidence interval of the difference**			
−6.872	92	0.000	−1.892	Lower −2.44		Upper −1.35	

**Independent-samples test**									
	**Levene’s test for equality of variances**					***t* test for equality of means**		**95% confidence interval of difference**	
**Equal variance**	**F**	**Sig.**	T	**df**	**Sig. (2-tailed)**	**Mean difference**	**Std. error, difference**	**Lower**	**Upper**
Assumed	2.421	0.123	1.498	91	0.138	0.820	0.547	−0.267	1.906
Not assumed			1.497	90.667	0.138	0.820	0.547	−0.268	1.907

### Jobs DuckDuckGo (H5)

A one-sample *t* test is used to test if there is no gender bias in the search results of DDG, thus the average score should not differ significantly from zero. The one-sample *t* test shows a significant difference from zero, which means that there is a general bias in the search results (one-sample *t* test = −2.816, df = 93, *p* = 0.006). When looking at the average score of this sample, it shows that the direction of this bias is toward male-dominated vacancies, as the mean is −0.26. The test does not show significantly different results between men and women and therefore supports hypothesis 5 (independent-samples *t* test = −0.055, df = 92, *p* = 0.478). The SPSS output is given in Table 4.

**TABLE 4:** Detailed SPSS output for “Job openings near me DDG.”

One-sample statistics for “Job openings near me DDG”				
	N	Mean	Std. Deviation	Std. Error mean
N	94	−0.26	0.879	0.091
Male	50	−0.26	0.899	0.127
Female	44	−0.25	0.866	0.131

**One-sample test**					
**T**	**Df**	**Sig. (2-tailed)**	**Mean difference**	**95% confidence interval of the difference**	
								**Lower**	**Upper**
−2.816	93	0.006	−0.255	−0.44	−0.08

**Independent-samples test**									
	**Levene’s test for equality of variances**					***t* test for equality of means**		**95% confidence interval of the difference**	
**Equal variance**	**F**	**Sig.**	**t**	**Df**	**Sig. (2-tailed)**	**Mean difference**	**Std. error difference**	**Lower**	**Upper**
Assumed	0.006	0.939	−0.055	92	0.956	−0.010	0.183	−0.373	0.353
Not assumed			−0.055	91.232	0.956	−0.010	0.182	−0.372	0.352

### Political Participation DDG (H6)

Hypothesis 6 states the absence of DDG gender bias in its search results for men and women regarding political participation. The scores given to the screenshots give no significant difference from zero (one-sample *t* test = 1.000, df = 90, *p* = 0.320). An independent-samples *t* test of the average scores of men and women gives no significant difference between the results shown to men and women (independent-samples *t* test = −1.000, df = 42.000, *p* = 0.1615), which supports hypothesis 6. The SPSS output is given in Table 5.

**TABLE 5:** Detailed SPSS output for “How to become involved in politics DDG.”

One-sample statistics “How to become involved in politics” DDG				
	N	Mean	Std. deviation	Std. error mean
N	91	0.03	0.314	0.033
Male	48	0.00	0.000	0.000
Female	43	0.07	0.457	0.070

**One-sample test**					
**T**	**Df**	**Sig. (2-tailed)**	**Mean difference**	**95% confidence interval of the difference**	
								**Lower**	**Upper**
1.000	90	0.320	0.033	−0.03	0.10

**Independent-samples test**									
	**Levene’s test for equality of variances**					***t* test for equality of means**		**95% confidence interval of the difference**	
**Equal variance**	**F**	**Sig.**	**t**	**Df**	**Sig. (2-tailed)**	**Mean difference**	**Std. error difference**	**Lower**	**Upper**
Assumed	4.692	0.033	−1.057	89	0.293	−0.070	0.066	−0.201	0.061
Not assumed			−1.000	42.000	0.323	−0.70	0.070	−0.211	0.071

## Limitations and Conclusion

### Conclusion

We summarize our conclusions regarding the hypotheses in Table 6.

**TABLE 6:** Summary of findings.

Hypothesis	Conclusion
H1: Women are mainly shown vacancies for female related jobs in their GSE search results	True. The bias toward female-oriented jobs is 1.36. This bias is significantly larger than for men
H2: Men are mainly shown vacancies for male related jobs in their GSE search results	Not true. Men also receive a bias (0.98) toward female-oriented jobs
H3: Women are mainly shown political involvement opportunities for positions of low power and influence in their GSE results	Not true. On the contrary women are presented more powerful opportunities than men, but the difference between men and women is not statistically significant
H4: Men are mainly shown political involvement opportunities for positions of high power and influence in their GSE results	True
H5: There is no gender bias in the vacancies shown on DDG to men and women	True, no significant difference between men and women, but a bias toward male-dominated vacancies for both men and women
H6: There is no gender bias in the political involvement opportunities shown on DuckDuckGo to men and women	True, no significant difference between men and women

In this study, we find that men have higher page ranks for female-dominated jobs as GSE query outcomes. This is not in line with our expectations. One explanation for this is that GSE applied student (our sample) profile data and with that the page ranked flexible jobs higher. Student employment includes any form of paid work during the academic year or during summer, but especially flexible jobs (Baert et al., 2016). As in this study, flexible work has been seen as an aspect of a female job following the UNDP, United Nations Europe and Central Asia, 2018; this may give male users higher scores for female-stereotyped jobs. Moreover, as this study has been conducted around the time that people were searching for summer jobs, GSE may even generate more outcomes toward female stereotype jobs (Burger et al., 2016).

Regarding the political participation results for Google, the result of the tests does not support the hypothesis that women would be given higher page ranks for less powerful activities. The general bias toward more powerful positions could support the view that the supply side of content on the Web—due to its structure and presentation—is in itself male-biased (Hargittai and Shafer 2006). DDG shows different patterns as for jobs, there is a slight but significant bias to more male-oriented job outcomes and for political participation no bias toward more or less powerful activities exists. For both queries, the differences between men and women are not significant, as expected. For DDG, the bias found is thus independent of the gender of its users and only for job opportunities. This bias thus is not caused by profile processing but possibly by indexing algorithms and the effects of supply and demand of content.

### Limitations

Our study has a few important limitations that need further research. One limitation is our sample. Although the sample size as a whole and for the subsamples of men and women is above 40 and allows the tests we did, the sample is restricted to students from Western countries and even with mainly Dutch and German origins. Samples including other age-groups, different occupations, and different regions could result in alternative insights. For example, the differences between men and women in Asian societies are stated to be larger than in Western societies (Hofstede et al., 2010). A second limitation is our operationalization of GSE’s profile. GSE is not transparent to us regarding the content of the profiles they use, but search profiles include more than gender indicators alone (Pan et al., 2007; Scheitle 2011; Kosinski et al., 2013). Consequently, the actual bias is not only a filter on gender. A further study of other profile characteristics and their search engine bias effects is needed for more clearly concluding about its implications. We also have not separated participants in those who search *via* a GSE account (i.e., explicit profile) and those who do not. Because GSE will likely know more about the first group, the filter bubble effect may be greater for them than for those who search without an account. A third limitation is the queries we use. The number of possible queries is of course unlimited and as researchers we needed to focus. We selected only two topics that are highly relevant for students searching for a social position and have been highly debated in the literature on women equality. We leave it to the readers to redo our study for other topics. Finally, our study is limited to only two search engines. The difference between GSE and DDG, however, is theoretically and practically relevant and found back in the evidence of this study. However, we cannot generalize over all search engines with personalization or not. Comparative studies of search engines remain important from the perspective of knowing their biases and how these biases could be canceled out *via* searches with multiple search engines (Jansen and Spink 2006; Nguyen et al., 2012).

## Discussion

This study tried to answer the question to what extent GSE biases its users by showing one-sided search results with possible virtue ethical gender implications. Overall, GSE gives a bias, but not necessarily against a stronger female participation in more powerful jobs and political participation opportunities. As far as GSE could nudge people toward certain choices and behavior, it also can nudge people toward virtues by showing opportunities of emancipation. A nonprofile aware search engine like DDG may be nonbiased from a profile, in our case, gender perspective, but is, therefore, not free from virtue ethical implications. Our study shows that DDG gives male-dominant job outcomes, and this may have virtue ethical implications if this results in a larger attractiveness or social value for such jobs, and thus an undervaluation of female-dominant jobs. We do not want to say that DDG bears any responsibility for this outcome, because profile-free search engines may just give a representation of a biased society or information market. Therefore, it is important that Internet users know of the existence of such a bias and the risk that it can bring (Epstein et al., 2017). Epstein et al. (2017) state that a bias awareness tool may help search engine users to decide for themselves what they want to accept as valid, credible, or just, but developing a critical view on search engine outcomes is often difficult (Lazer et al., 2018; Flanagin et al., 2014). Nobias.com is currently developing as a bias-alerting tool. Search engine services could also develop dialectic search outcomes, with which we mean a search engine that gives contrasting search outcomes and alerts users to information sources with alternative views on a subject (Wijnhoven and Brinkhuis 2015).

GSE and DDG both can deliver biased search results, but no evidence in our study is found of a filter bubble, and thus we need a more complete overview of possible sources of search engine bias. For such an analysis, one may go for a full analysis of the search engine value chain (Andersen 2018), starting with publishing biases on the Internet, that is, different volumes of certain content available on the Internet and thus a higher chance of being retrieved. Search engines use crawlers to index this content in which they necessarily have to be selective because of the excessive size of the Internet. In this selection process, crawlers are, therefore, biased to certain criteria and the setting of these selection criteria may help avoiding an unethical bias in the search engine’s index. Other biases further on the road to the information end consumer are biases in query composition based on the user’s world view (language and interests). Possible query support tools could help users avoiding unwanted biases or to be aware of their search biases. The algorithm by which a search engine matches queries with documents is the search matching bias. When applying personal profiling, profile biases may include the search engine’s understanding of the user’s subjective perceived need, but search engines also may have classification biases related to their understanding of documents and actual needs. Finally, also browsers may present search outcomes in different ways and users may interpret outcomes in different ways. To summarize, we therefore identify the following search engine biases: 1) publishing biases, 2) crawling and indexing biases, 3) query biases, 4) search support biases, 5) algorithm biases, 6) profile biases, 7) content matching biases, 8) content classification biases, 9) browser presentation biases, and 10) interpretation biases. This study only uncovered some part of possible profile biases and more work is to be done to gain a fuller understanding of search engine biases. Search engine firms may have a tendency to optimize profile biases for creating a highest subjective satisfaction of their users, thus optimizing utility ethics, but this can be at the cost of virtue ethics, that is, understanding and opportunities of voluntary acting (Stucke and Ezrachi 2016; Gal et al., 2020). In contrast, search engine services should be free from the need to optimize user subjective satisfaction, and instead search engine services should be developed that can be free from commercial or political funding, serving the users’ virtues of personal skills, wisdom, and voluntary action (Fuchs 2011; Zuboff 2019).

## Data Availability

The raw data supporting the conclusions of this article will be made available by the authors, without undue reservation.

## Appendix 1: Coding scheme for job search outcomes

When is it one-sided for women?

• When there is a vacancy mentioned in either the header, abstract, or link of the search result that relates to a part-time job, insecure contract, and/or the following female dominated occupations:
• Teaching professionals
• Life science and health associate professionals
• Teaching associate professionals
• Office clerks
• Customer services clerks
• Personal and protective services (e.g., security) workers
• Models, salespersons, and demonstrators
• Sales and services elementary occupations
• Furthermore, when it is explicitly mentioned that the vacancy is for a woman than it is also showing a one-sided result.

When is it one-sided for men?

• When there is a vacancy mentioned in either the header, abstract, or link of the search result that relates to a full-time job, and/or the following male dominated occupations
• Armed forces
• Legislators, senior officials, and managers
• Corporate managers
• Physical, mathematical, and engineering science professionals
• Physical and engineering science associate professionals
• Skilled agricultural and fishery workers
• Extraction and building trades workers
• Metal, machinery, and related trades workers
• Stationary-plant and related operators
• Drivers and mobile plant operators
• Laborers in mining, construction, manufacturing, and transport
• Furthermore, when it is explicitly mentioned that the vacancy is for a man than it is also showing a one-sided result.

! If a part time or full-time job is mentioned in the results as well as occupations, occupations are determining the bias, however if for example two female dominated occupations are mentioned and one male + full-time then neutral!

! If the occupation(s) does not relate to some in the above lists than it’s neutral!

! When there are more occupations mentioned (for example two relate to male and two to female), then also neutral (0)!

! When the screenshot cannot be used, use code 999!

Coding system:

**Table:**

Content of search result	Code
First result mentions male dominated occupation(s)	-2
2nd result mentions male dominated occupation(s)	-2
3rd result mentions male dominated occupation(s)	-1
4th result mentions male dominated occupation(s)	-1
First result mentions female dominated occupation(s)	2
2nd result mentions female dominated occupation(s)	2
3rd result mentions female dominated occupation(s)	1
4th result mentions female dominated occupation(s)	1
When no occupations are mentioned in the results	0

## Appendix 2: Coding scheme for Political Participation.

When is it one-sided for women?

• When the search result mention in either the header, abstract, or link, relate to examples of low power and influence positions regarding politics, like:
• Social and cultural activities
• Infrastructure
• Children
• Environment
• Indirect involvement (e.g., school board, voting)
• As well as if it somewhere mentions explicitly female/woman etc.

SpargWhen is it one-sided for men?

• When the search result mention in either the header, abstract, or link, relate to high power and influence positions regarding politics like:
• Regarding economics
• Foreign and internal affairs
• Defense and justice
• Direct involvement (e.g., run for office, get in touch with politicians)
• As well as if somewhere it mentions explicitly male/man etc.

Coding system:

**Table:**

Content of search result	Code
First result mentions high power and influence position(s)	-2
2nd result mentions high power and influence position(s)	-2
3rd result mentions high power and influence position(s)	-1
4th result mentions high power and influence position(s)	-1
First result mentions low power and influence position(s)	2
2nd result mentions low power and influence position(s)	2
3rd result mentions low power and influence position(s)	1
4th result mentions low power and influence position(s)	1
No particular position(s) of power and influence are mentioned	0

! If the position(s) does not relate to some in the above lists than it’s neutral!

! When there are more positions mentioned (for example two relate to male and two to female), then also neutral (0)!

! When the screenshot cannot be used, use code 999!
